# Effects of In-Season Strength Training on Physical Fitness and Injury Prevention in North African Elite Young Female Soccer Players

**DOI:** 10.1186/s40798-024-00762-0

**Published:** 2024-09-02

**Authors:** Manel Darragi, Hassane Zouhal, Mariem Bousselmi, Houssem M. Karamti, Cain C. T. Clark, Ismail Laher, Anthony C. Hackney, Urs Granacher, Amira B. M. Zouita

**Affiliations:** 1 Higher Institute of Sport and Physical Education of SFAX University of Sfax, Sfax, Tunisia; 2 Research Unit (UR17JS01) “Sport Performance, Health & Society”, Higher Institute of Sport and Physical Education, Ksar Said, Tunisia; 3 Movement, Sport, Health and Sciences Laboratory (M2S). UFR APS, University of Rennes 2-ENS Cachan, Av. Charles Tillon, 35044 Rennes Cedex, France; 4 Institut International Des Sciences du Sport (2I2S), 35850 Irodouer, France; 5 College of Life Sciences, Birmingham City University, Birmingham, B15 3TN UK; 6 Department of Anesthesiology, Pharmacology and Therapeutics, The University of British Columbia, Vancouver, Canada; 7 Department of Exercise and Sport Science, University of North Carolina, Chapel Hill, NC USA; 8 Department of Sport and Sport Science, Exercise and Human Movement Science, University of Freiburg, Freiburg, Germany; 9 Higher Institute of Sport and Physical Education of Ksar Said, University of Manouba, Tunis, Tunisia

**Keywords:** Resistance training, Football, Adolescent, Performance, Women

## Abstract

**Background:**

Strength training (ST) primarily enhances physical fitness (e.g., muscle strength, power, speed) and bone density in female soccer players. Less information is available on the injury preventive effects of ST in female athletes. Accordingly, this study aimed to investigate the effects of a 12-week in-season ST on measures of physical fitness and injury occurrence in young elite female soccer players.

**Methods:**

Thirty elite female soccer players (15.4 ± 1.9 years; maturity offset + 2.3 ± 1.1 years) participated in this study, and were randomly assigned to a strength training group (STG, n = 14) or an active control (CG, n = 16) group. ST lasted twelve weeks and included full body muscle strengthening exercises using primarily weight machines at progressive intensities ranging between 40 and 85% of the one-repetition-maximum (1-RM). The CG practiced a traditional soccer training program. Overall, training volumes of the two groups were similar with a training load (rating of perceived exertion × time) of 1158.4 ± 67.7 arbitrary unity (AU) for the STG and 1188.8 ± 44.1 AU for the CG. Pre and post training, the following physical fitness tests were applied: dynamic muscle strength (relative [to body mass] and absolute 1-RM bench/leg press, lat-pull down), jump performance (countermovement jump [CMJ], squat jump [SJ], five-jump-test [5JT]), linear-sprint speed (5-m, 10-m, 30-m), change-of-direction speed (T-test with and without ball), sport-specific performance (Yo-Yo Intermittent Level1 [YYIRTL1], and repeated shuttle sprint ability [RSSA]). The injury rate per 1000-h exposure was monitored throughout the soccer season.

**Results:**

No significant baseline differences were observed between groups. Statistically significant group-by-time interactions were found for absolute (*p* < 0.001, d = 2.59) and relative 1-RM bench press (*p* < 0.001, d = 2.39), absolute 1-RM lat-pull down (*p* < 0.001, d = 1.68), and relative 1-RM leg press (*p* < 0.001, d = 1.72). Significant group-by-time interactions were observed for CMJ (*p* = 0.005, d = 1.27), RSSA_mean_ (*p* = 0.007, d = 0.81), and RSSA_total_ (*p* < 0.001, d = 1.90). Post-hoc tests indicated that the STG group demonstrated greater improvements in all tested variables compared to CG (1.2 < d < 2.5). However, no significant interaction effects were noted for measures of linear sprint speed and YYIRTL1 performance. Additionally, non-contact injuries during the season were significantly lower (*p* = 0.003, d = 1.31) in the STG (0.48/1000 h of exposure) than the CG (2.62/1000 h of exposure).

**Conclusions:**

Twelve weeks of an in-season ST resulted in larger physical fitness improvements and fewer injuries compared with an active control in elite young female soccer players. Accordingly, ST should be systematically applied in female soccer to enhance performance and prevent injuries.

## Key Finding

- This study showed that the incorporation of 12 weeks of strength training with two weekly sessions during the soccer season improves anthropometric and body composition characteristics as well as measures of physical fitness in adolescent elite female soccer players.
- Strength training reduces the incidence of non-contact injuries in young female soccer players.
- The usage of strength training machines is safe in elite young female soccer players with no previous experience in weightlifting.

## Introduction

Soccer is one of the most popular sports throughout the world, and its broad appeal and popularity continue to grow [1]. Soccer is played by males and females of different expertise and age levels [2]. Understanding the specific requirements of elite-level soccer players can provide information about the factors that contribute to improved performances in competitions. The maintenance of high physical fitness levels across the season and the concomitant avoidance of injuries are key for successful soccer team performance [1].

Female soccer continues to experience significant growth in terms of market share, resulting in more opportunities to play professionally. Between 2010 and 2015, the number of females (girls and grown-ups) has increased by 32% and reached a global number of 30 million female soccer players [3]. The “Fédération Internationale de Football Association” (FIFA) estimates that female soccer participation will double to 60 million worldwide by 2026 [2, 4]. Moreover, the availability to train and gain exposure and match play is important for high-performance young female soccer players to develop physically and to improve their technical and tactical skills [4] leading to a growth of academic structures to nurture the development of young players at various expertise levels [5].

Match play analysis revealed that particularly in female soccer, matches became more intense in terms of running distance and intensity compared to previous tournaments [6]. Accordingly, a larger focus should be laid on physical fitness development in young female players to set the ground for later high-intensity performance during match play. Female professional soccer clubs are currently investing their resources in creating youth soccer academies to systematically nurture and develop future professional female soccer players [4]. Besides the need to further increase physical fitness of female soccer players to cope with the increased match play demands, there is also evidence indicating five-time higher injury rates in female versus male soccer players, particularly related to anterior cruciate ligament (ACL) injuries [7, 8]. This increased vulnerability to injuries could be related to anatomical (e.g., wider pelvis), biomechanical (e.g., knee valgus), hormonal, and/or neuromuscular factors [7]. Further, Mandorino et al. [9] specified that male soccer players are more prone to muscle strains and ligament sprains, while female players suffer more from ligament sprains.

Sustaining an injury reduces a player's availability and can negatively influence future performance by reducing fitness and harming developmental progression and future career opportunities [1]. The repercussions of injuries on the development and well-being of players require an improved understanding of the incidence, prevalence, and severity of injuries in elite young soccer so that appropriate programs aimed at reducing injuries can be implemented [10].

Recent studies indicated that well-designed strength and conditioning programs are key for female athletes to systematically develop physical fitness [11] and prevent injuries [12]. Various intervention programs have been introduced to improve physical fitness, including neuromuscular training, plyometric training, strength training (ST), and combinations of ST with other exercises [12]. However, there is a lack of consensus on the most effective exercise program to improve physical fitness and reduce injuries in female soccer players [11].

Hormonal variations during the menstrual cycle are associated with injury susceptibility in female athletes, as indicated by studies showing that fluctuations in female sex hormones, such as estrogen and progesterone, influence various physiological factors that can contribute to an elevated risk of sustaining injuries in female athletes [13].

Engaging in ST programs may have prolonged positive effects on young female soccer players by improving various physical attributes essential for performance and injury prevention. When implemented consistently, these programs lead to improvements in muscle strength, power, speed, agility, endurance, and reduce injury risks over time. More specifically, a group of researchers examined under-19 elite male soccer players and demonstrated that a ST program conducted twice a week for 10 weeks reduced the total number of injuries and hamstring injuries. The injury rate ratio (IRR) was lower during the study period, with a substantial reduction in injury incidence. Furthermore, there was a reduction in the number of absence days associated with each injury and in the number of absence days per 1000 h during the study. The authors concluded that the training program had long-term benefits in preventing muscle injuries in soccer players [14]. In terms of the long-term effects of ST, another study [15] demonstrated that young female soccer players benefited from well-designed strength/power and complex training programs leading to sustained improvements in physical fitness, ball-shooting performance, and a reduced incidence of injuries over time [15]. This phenomenon can be explained because ST induces improved neuromuscular function leading to better motor coordination, the strengthening of adjacent tissues, reducing critical joint loads, and increasing psychological perception of high-risk situations [16–18].

The increase in training load and competitiveness combined with incomplete muscle mass development can predispose youth athletes to muscle strain. In fact, the growth spurt period is a critical time for young athletes when bone and soft tissue development could lead to reduced flexibility and, in turn, growth-related injuries [4, 9]. In contrast, young female soccer players appear to be more prone to ligament sprains than to muscle strains [9]. Accordingly, it is important to investigate whether ST programs have the potential to reduce injury rates in female soccer players [6].

Our experience indicates that coaches working with athletes at an early age devote more time to improve their players' technical and tactical characteristics, resulting in limited time for strength and conditioning programs. Anecdotal evidence from coaches of North African female soccer teams indicate that ST is seldomly included in the in-season soccer training, particularly in youth but also elite female soccer. Furthermore, to the authors’ knowledge, there is no study available that has examined the impact of in-season ST on measures of physical fitness and non-contact injuries in North African elite young female soccer players.

Accordingly, this study examined the effects of a 12-week ST on soccer-performance related measures of physical fitness and the occurrence/recurrence of injuries in young elite female soccer players. Based on the available literature [14, 15, 17, 18], we hypothesized that a 12-week ST program with two weekly sessions, each lasting 30 min improves measures of physical fitness and reduces the incidence of non-contact injuries, particularly related to muscle strains and ligaments.

## Methods

### Participants

A minimum sample size of 18 was determined from an a priori statistical power analysis using G*Power (version 3.1, University of Düsseldorf, Germany) and the primary endpoint physical fitness development (linear sprint speed). The power analysis was calculated with an assumed power of 0.90 at an alpha level of 0.01, a non-sphericity correction of 1, and a moderate effect size of 0.38 taken from a related study [19] for the primary endpoint 10-m linear sprint speed. Twenty-nine elite female soccer players from the Tunisian U15 national team (age = 15.4 ± 1.9) were randomly allocated to an experimental group (STG, n = 14) or an active control group (CG, n = 16) (Table 1). Prior to study participation, players did not systematically practice ST. The maturity status was determined using the maturity-offset method according to Mirwald et al. [20].

**Table 1:** Characteristics of study participants at baseline

Groups	Age (years)	Body mass (kg)	Bod Height (cm)	BMI	PHV	Body fat (%)
CG (n = 16)	15.5 ± 0.9	57.8 ± 7.7	163.9 ± 6.6	21.5 ± 2.4	2.6 ± 1	26.9 ± 2.6
STG (n = 14)	15.2 ± 0.9	55.2 ± 8.6	163.9 ± 7.5	20.3 ± 2.9	2.1 ± 0.6	25.4 ± 3.4

*CG* control group, *STG* strength training group, *BMI* body mass index, *PHV* peak height velocity

All players followed the same training regime provided at an elite youth soccer academy. The enrolled participants exercised nine months per year, with five weekly sessions, each lasting 90 min, and a match on the weekend. In addition to soccer training, players from both groups performed two physical education lessons per week, each lasting one hour. It is important to note that all players were part of the soccer academy during the soccer season, except for weekends when they returned to their families or joined the soccer team to play a match. Both groups (STG and CG) followed the same nutrition and hydration protocols during the soccer season. Dietary and hydration habits were managed by the soccer academy’s nutrition staff, and nutritional guidelines were provided to athletes for weekend meals.

All individuals were familiarized with the experimental protocol, and players and their parents provided informed consent before study participation. Our study was approved by the local ethics committee of the University of Sfax, Sfax, Tunisia (C.P.P.SUD No 0494 /2023) following the latest version of the Declaration of Helsinki and the medical ethics committee of the Tunisian Football Federation.

### Selection Criteria

To be eligible for inclusion, participants had to be female elite soccer players aged under 15 years without any acute pathologies or injuries before or during the experimental study period. Participants were excluded if they suffered from any acute pathology or injury before or during the study period or if they already performed systematic ST, received other physio-therapeutical or pharmacological treatment. Players with a participation rate of less than 85% during training sessions were excluded from the study. A total of 26 players completed all pre and post-tests with four players being injured (three from the CG and one from the STG) prior to post-tests.

### Procedures

Anthropometrics and body composition were collected for all participants. Physical fitness tests comprised absolute and relative (relative to body mass) maximal dynamic muscle strength (i.e., one repetition maximum [1-RM] leg press, 1-RM bench press, 1-RM lat pull-down), muscle power (i.e., squat jump [SJ], countermovement jump [CMJ], five jump test [5JT]), linear sprint speed (5-m, 10-m, 30-m distances), change-of-direction (CoD) speed (T-test with and without ball), soccer-specific performance (Yo-Yo Intermittent Level1[YYITL1], repeated shuttle sprint ability test [RSSA]).

An active recovery of at least two minutes was provided between each fitness test where participants engaged in low-intensity activities such as walking or ball passing to sustain their readiness for the subsequent test. Before the tests started, a warm-up consisting of ten minutes of general exercises, including submaximal running with CoD, and specific exercises both vertical and horizontal submaximal jumps, was conducted. Furthermore, participants executed test-specific warm-ups, consisting of two familiarization jumps or runs.

**Fig. 1:**
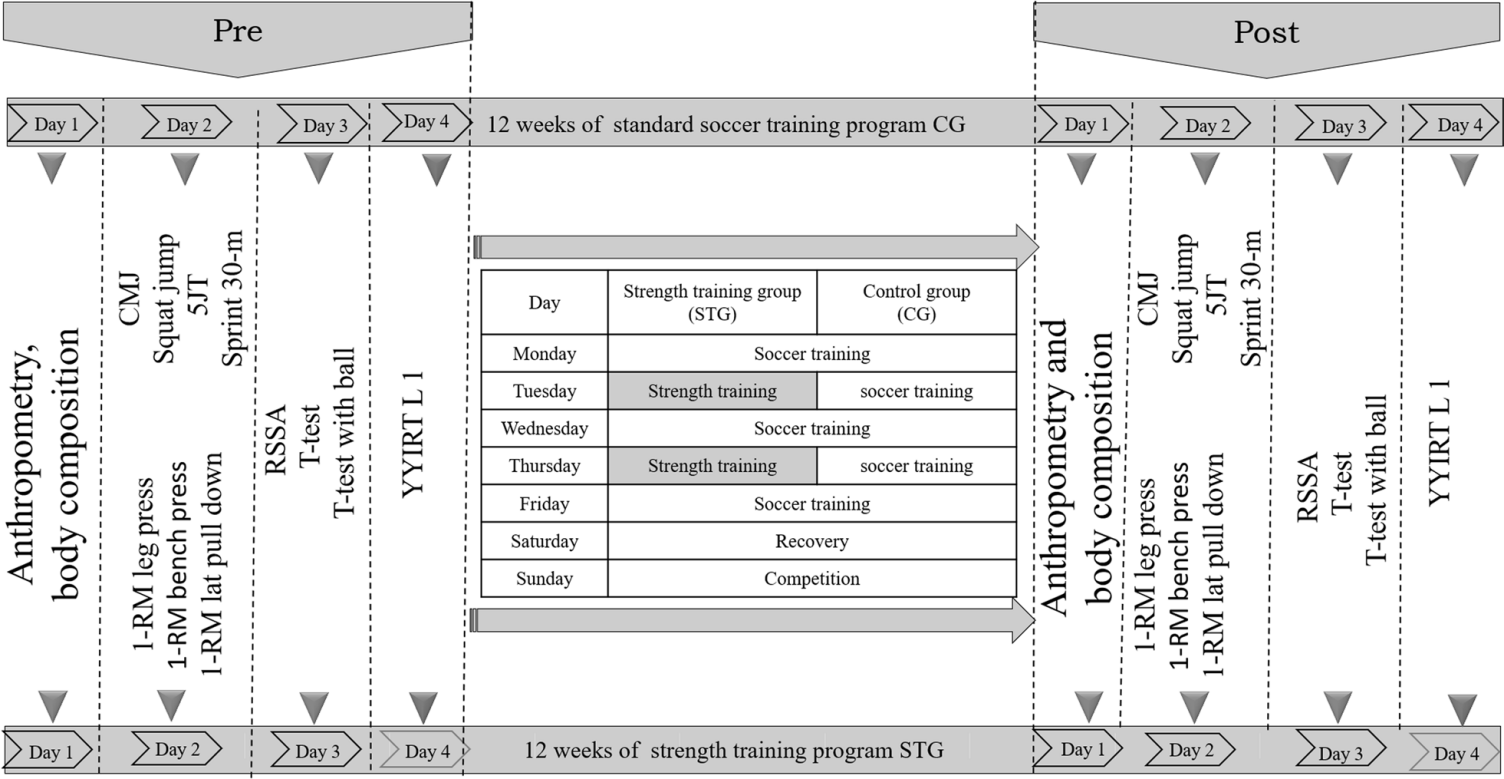
Study flowchart. CMJ, countermovement jump; SJ, squat jump; 5JT, five-jump test; RSSA, repeated shuttle sprint ability; YYIRTL1, yoyo intermittent recovery test level1; 1-RM, one repetition maximum; STG, strength training group; CG, control group

The same assessors supervised all pre-post test procedures and were blinded to group allocation. All participants were familiar with all test procedures. The same order and the same time of day (between 4:00 pm and 5:30 pm) were used for all tests (Fig. 1).

### Training Program

The 12-weeks in-season ST was conducted in three cycles from December to February, with each cycle lasting four weeks (Table 2). The first cycle comprised three weeks of ST followed by one week without ST but only soccer practice. During the three ST weeks, STG practiced five exercise sessions per week (two ST and three soccer-specific sessions) while the CG continued with their five sessions of regular soccer training. Accordingly, STG performed the same contents during soccer training but replaced 90 min of soccer training twice weekly with ST. ST was conducted on non-consecutive days with a 48 h rest between the two ST sessions to ensure adequate recovery. Progression was achieved by increasing the training intensity over the course of the study. The first cycle focused on a strength endurance program at 40–60% of the 1-RM using slow movement speeds during exercise performance. The program started with bodyweight exercises to master the technique before incorporating additional weights using weight machines. During the first cycle, participants performed three sets of 15 repetitions for each ST exercise. The emphasis of the second cycle comprised exercise intensities in the range of 60–75% of the 1-RM at slow movement speeds. As the load was increased from the first to the second cycle, the number of repetitions consisted of three sets with 12–10–8 repetitions for each set. Finally, for the third cycle, exercise intensity was increased to 85% of the 1-RM. Players performed three sets per exercise and 10–8-6 repetitions per set. The 1-RM was assessed every four weeks and the elements of the ST were adjusted accordingly.

**Table 2:** Exercise programming during the 12 weeks strength training intervention

Parameters	Week 1	Week 2	Week 3	Week 4	Week 5	Week 6	Week 7	Week 8	Week 9	Week 10	Week 11	Week 12
% RM	BW	40% 40% 45%	45% 55% 60%	Recovery	45% 55% 60%	55% 60% 65%	65% 70% 75%	Recovery	65% 70% 75%	70% 75% 80%	75% 80% 85%	Recovery
Nb repetition	15	15	15		12 10 8	12 10 8	12 10 8		10 8 6	10 8 6	10 8 6	
Nb series	3	3	3		3	3	3		3	3	3	
	30"	45"	1′30''		2'	3'	3'		3'	3'	3'	
Exercise selection												
Lower limbs												
Leg exercises												
Body mass—box squat	X											
Body mass—squat		X										
Smith machine—back box squat			X		X							
Smith machine squat						X	X		X	X	X	
Split squat	X											
Hip thrust	X	X	X		X	X	X		X	X	X	
Leg press		X	X		X	X	X		X	X	X	
Standing calf raise			X		X	X	X		X	X	X	
Upper limbs												
Push exercises												
Floor push up	X											
Kneeling push up		X										
Smith machine bench press			X		X	X	X		X	X	X	
Pull exercises												
Box static pull up	X	X										
Lat pull down wide pronated grip			X		X	X	X		X	X	X	
Trunk												
Core exercises (warm-up)												
Kneeling front plank	X											
Kneeling side plank	X											
Front plank		X				X						
Side plank		X										
Front plank—hip extension		X					X		X	X	X	
Y dorsal raise hold	X	X							X	X	X	
Dead bug			X		X							
Press pallof			X									
Bridge: static			X									
Nordic hamstring					X	X	X		X	X	X	
Kneeing side plank with hip abduction					X	X	X		X	X	X	
Reverse plank					X	X	X					

*RM* repetition maximum, *BW* body weight, *Nb* number

A 15-min warm-up was performed before each exercise session. A five-minute cool-down was scheduled after the training including dynamic stretching exercises. ST was supervised throughout the study period by experienced strength and conditioning coaches.

### Anthropometry and Body Composition

Body mass was assessed on the Beurer brand impedance-meter scale (Beurer GmbH, BF180, Ulm, Germany) to the nearest 0.1 kg. Standing and sitting height by using a stadiometer at the nearest 0.1 cm and the lower-limb length by a measuring tape.

To assess body fat, skinfold thickness was measured at four specific locations on the left side of the body (triceps brachii, biceps brachii, subscapular, and suprailiac) using a skinfold caliper (Harpenden, British Indicators Ltd., Luton, UK). Body fat percentage was calculated using the formula outlined by Durnin and Womersley [21]. Lean body mass was calculated by using the Formula of Boer, (1984) [22]. For females:

LBM (kg) = (0.252 × weight [kg]) + (0.473 × height [cm]) − 48.3

The intra-class correlation coefficients (ICC) for test–retest reliability and the coefficient of variation (CV) of the anthropometric measures are presented in Table 3.

**Table 3:** Intraclass correlation coefficients (ICCs) for relative reliability and coefficients of variation (CVs) for absolute reliability of the applied anthropometric tests

Anthropometric and body composition tests			
Measures	ICC	95% CI	% CV
%Body fat	0.932	0.848–0.969	4.47
Body mass	0.989	0.976–0.995	3.09
Height	0.997	0.993–0.999	0.38
BMI	0.984	0.965–0.993	3.13
Sitting height	0.951	0.890–0.978	1.11
Leg length	0.987	0.972–0.994	1.01

*ICC* intraclass correlation coefficient, *CI* confidence interval, *CV* coefficient of variation (%), *BMI* body mass index

### Maturity Status

We gauged maturity (i.e., years from peak height velocity [PHV]) using standing and sitting height according to the approach as introduced by Mirwald et al. [20].

Maturity offset (PHV) = − 9.236 + (0.0002708 × leg length × sitting height) − (0.001663 × chronological age × leg length) + (0.007216 × chronological age × sitting height) + (0.02292 × (body mass/body height × 100).

This equation has been previously employed to assess maturation in young female soccer players [2].

### Physical Fitness Tests

Field assessments were conducted pre- (T1) and 12 weeks post-intervention (T2) to evaluate the effects of ST on the physical fitness of female soccer players. Both T1 and T2 were performed on a 4th generation tartan grass soccer field under the same environmental conditions (~ 22 °C and ~ 36% humidity) (local meteorological service data). Participants used the same soccer sports clothes during pre and post tests that they usually wore during training and competitions.

#### Maximal Dynamic Strength

The maximal dynamic strength tests performed in our study were 1-RM bench press, 1-RM lat pull-down, and 1-RM leg press using weight-lifting machines (Matrix, Houdan, France). The 1-RM was determined according to guidelines recommended by the American College of Sports Medicine (ACSM) [23].

After a general dynamic warm-up, the tests started with specific submaximal exercises consisting of three lifts with progressively increasing loads (40%, 75%, and 85% of 1-RM expected). The number of repetitions decreased as the session progressed (10 → 6 → 3). The first attempt was made with a load approximately 5% below the expected 1-RM. If a lift was successful, the load was increased by approximately 5%, and the test ended when the player failed to lift the load after 2–3 attempts. The maximum load successfully lifted was noted as 1-RM. Players rested for three minutes between lifts [24].

The ICC for test–retest reliability and the CV for the maximal dynamic strength tests are presented in Table 4.

**Table 4:** Intraclass correlation coefficients (ICCs) for relative reliability and coefficients of variation (CVs) for absolute reliability of the applied maximal dynamic strength tests

Maximal dynamic strength tests			
Measures	ICC	95% CI	% CV
1-RM absolute lat pull down	0.648	0.215–0.842	14.35
1-RM relative lat pull down	0.860	0.567–0.913	12.37
1-RM absolute bench press	0.862	0.691–0.938	20.33
1-RM relative bench press	0.893	0.761–0.952	17.53
1-RM absolute leg press	0.864	0.698–0.939	20.40
1-RM relative leg press	0.844	0.652–0.930	20.98

*ICC* intraclass correlation coefficient, *CI* confidence interval, *CV* coefficient of variation (%), *1-RM* one repetition maximum

#### Muscle Power

Each participant executed two types of maximal jumps: the SJ and the CMJ. The jump height produced during these vertical jump tests were assessed using an optoelectric system (Opto Jump Microgate, Bolzano, Italy) and calculated using the following formula:

Jump height = 0.125 × g × t^2^ where g is the gravitational acceleration and t is the flight time or duration [17].

Players also performed the 5JT [25]. Three trials were conducted, with a 1-min rest between each, and the best-performing trial was selected for subsequent statistical analysis.

#### Squat Jump (SJ)

During the SJ, participants were directed to place their hands on their hips, stand with their feet shoulder width apart, and start the test in a flexed knee position (approximately 90 degrees). After a standardized test instruction (perform the test at maximal effort), participants accelerated in vertical direction. During flight time, the test instructor particularly spotted players’ test performance to make sure that they did not bend their knees which would prolong flight time and artificially enhance jump height. If knees were bended during the flight time, players had to repeat the test trial [23].

#### Countermovement Jump (CMJ)

The CMJ was employed to evaluate maximal vertical jump height performance, involving a slow stretch–shortening cycle (SSC) action. During the CMJ, participants were instructed to place their hands on their hips and stand with their feet shoulder width apart. From an upright erect stance, players performed a downward movement followed by a maximal effort vertical jump. Again, players were instructed not to bend their knees during the flight time [23].

#### Five Jump Test (5JT)

This test was conducted on grass surface, with participants wearing appropriate soccer boots. The 5JT involves five consecutive strides with the feet side-by-side at both the start and end of the jumps. Starting from the joined feet position, participants were not allowed to take any backward steps with either foot; instead, they had to directly jump forward using their preferred leg. After the initial four strides, alternating between left and right feet each time, the participants executed the last stride and concluded the test with both feet together. If the player fell backward upon completing the last stride, the test was repeated [25]. 5JT performance was measured with a tape measure starting from the front edge of the player’s feet in the starting position to the rear end of the feet in the final position.

#### Linear Sprint Speed

Sprint times were measured using double-light electronic gates (WITTY; Microgate, Bolzano, Italy), which were positioned at the start line (0 m) and 5, 10, and 30-m approximately 0.5 m off the ground. Participants initiated the test with one foot positioned 15 cm behind the starting line, standing in an upright, erect position, and were instructed to accelerate as fast as possible [23].

The interval between individual sprint trials ranged from 3 to 5 min, and the trial with the fastest sprint time from two attempts was selected for statistical analysis.

#### Change-of-Direction Speed (CoD)

The T-test was used as a CoD test and performed according to the modified Semenick procedure [26]. Participants conducted two trials, aiming for maximum effort. The trial with the fastest sprint time from the two trials was selected for data analysis. A 5-min rest interval separated each trial. T-test times (with and without ball) were recorded using the same photocell system used in speed testing.

#### Repeated Shuttle Sprint Ability (RSSA)

Following a standardized 15-min warm-up, players executed six sets of 20-m sprints (20 m outward and 20 m back) at maximum speed, with each sprint separated by 20 s of passive recovery. Sprint times were recorded using the same photocell system as employed in previous tests, and the best time was selected for result analysis (RSSA). Subsequently, the percentage decrease in performance, termed RSSA decrement, was calculated using the formula [27]:

$${\text{RSSA}}_{{{\text{decrement}}}} \, = \,\left( {\left[ {\text{average RSSA}} \right]/\left[ {\text{better RSSA}} \right]\, \times \,{1}00} \right)\, - \,{1}00)\,[27]$$

#### Yo-Yo Intermittent Recovery Test 1 (YYIRTL1)

The Yo-Yo intermittent recovery test 1 (YYIRTL1) consists of two 20-m shuttle runs at increasing speeds controlled by an audio metronome and interspersed with 10-s periods of active recovery until the individual is unable to maintain the required speed at a particular level [28].

The score recorded was based on the total distance covered on the last complete shuttle run.

The ICC for test–retest reliability and the CV for the physical fitness tests are presented in Table 5.

**Table 5:** Intraclass correlation coefficients (ICCs) for relative reliability and coefficients of variation for absolute reliability of the applied physical fitness tests

Physical fitness tests			
Measures	ICC	95% CI	% CV
5JT	0.780	0.501–0.903	13.64
SJ	0.581	0.065–0.812	11.99
CMJ	0.661	0.244–0.848	11.21
Sprint 5-m	0.700	0.330–0.865	5.13
Sprint 10-m	0.842	0.647–0.929	3.30
Sprint 30-m	0.873	0.716–0.943	2.85
T-test with ball	0.832	0.625–0.925	8.40
T-test without ball	0.856	0.679–0.935	5.22
YYRTL1	0.816	0.589–0.917	4.73
RSSA_Total_	0.960	0.911–0.982	1.59
RSSA_Best_	0.593	0.093–0.818	4.82
RSSA_mean_	0.832	0.625–0.925	4.03
RSSA %_decrement_	0.618	− 0.376–0.733	17.02

*ICC* intraclass correlation coefficient, *CI* confidence interval, *CV* coefficient of variation (%), *5JT* five-jump test, *SJ* squat jump, *CMJ* countermovement jump, *YYIRTL1* yoyo intermittent recovery test level1, *RSSA* repeated-shuttle-sprint ability

### Training Load Quantification

#### Internal Training Load

The assessment of the internal training load involves a modified version of Borg's rating of perceived exertion (session-RPE, sRPE) adjusted on a scale of 0 to 10 for the entire overall entire session. The sRPE was determined by multiplying the RPE for the training session with the duration of the session.

The equation [sRPE] = RPE x time is expressed in arbitrary units (AU) [29].

### Estimation of the Menstrual Cycle Phases

We used the calendar-based counting method to determine menstrual cycles since we could not assess serum estrogen (E2: 17-estradiol) and progesterone (P4) concentrations weekly [30]. The onset of each menstrual cycle (day 1 of menses) of the female soccer players was recorded monthly by the researcher staff. For the initial three consecutive menstrual cycles, we calculated each participant’s mean cycle length as the number of days before menstruation onset. A period of not more than 3 days standard deviation of each cycle length was considered to indicate a regular ovulatory menstrual cycle [31]. Participant injuries were evaluated according to the methodological recommendation of Janse De Jonge et al. [30], and menstrual cycles were divided into the early follicular phase (days 1 to 4), late follicular phase (days 10 to 13), and mid-luteal phase (days 20–23) based on a 28-days mean cycle.

### Assessment of Injury Rates Across the Soccer Season

Medical staff reported and validated each injury [32]. Injury type, location, and severity were recorded. Researchers reviewed the database every week and recorded individual player participation in both training sessions and matches [18]. These recorded injuries included any incident leading to a player's inability to fully participate in training or matches and were defined as time-loss injuries. A player remained classified as injured until the team's medical staff permitted full training and declared the player available for match selection. The injury monitoring continued throughout the soccer season.

#### Location of Injuries

We identified 7 specific locations to identify affected areas: foot, ankle, lower leg, knee, thigh, hip/groin and lumbar/sacrum/pelvis, [18, 32].

#### Type of Injuries

According to the Football Consensus Statement [32], soccer injuries are divided into seven groups: fractures and bone stress, joints (non-osseous) and ligaments, muscles and tendons, contusions, tears and skin injuries, central/peripheral nerves and other injuries. The severity of each injury is determined by the number of days from the date of injury to the player's full return to team training or competition. Injury severity was classified according to previous studies [18, 32] as minimal (≤ 3 days), mild (4–7 days), moderate (8–28 days), and severe (> 28 days). Recurrent injuries, defined as injuries of the same type and location that occur after a player has recovered and returned to full playing activity, have also been documented [32].

#### Calculation of Training and Match Exposure, Injury Incidence Rate and Burden

The number of injuries was recorded during training and matches and the incidence injury rate (IIR) and days lost (burden) per 1000 h were calculated using these equations:

$${\text{IIR }} = { 1}000 \, \times \, \left( {\sum {\text{ injuries}}/\sum {\text{ exposure hours}}} \right) \, \left[ {{32}} \right].$$

$${\text{Burden }} = { 1}000 \times \, \left( {\sum {\text{ days absent}}/\sum {\text{ exposure hours}}} \right) \, \left[ {{32}} \right].$$

## Statistical Analyses

Results are displayed as means and standard deviations (SDs). The statistical analysis was performed using SPSS software (SPSS, version 22, Chicago, IL) for Windows. The main statistics of the sample (mean and standard deviation) were obtained for all the sample variables and according to the group. The Shapiro–Wilk test was used to analyze the distribution of the sample in the two study groups.

Two-factor analysis of variance (ANOVA) (2 [groups: STG, CG] × 2 [time: pre, post]) was used to analyze within and between group comparisons over the twelve weeks of training. If a significant group-by-time interaction effect occurred, Bonferroni-adjusted post-hoc tests were computed. The results of the F test depended on the significance of Mauchly’s sphericity test. If significant, the Greenhouse–Geisser correction was used. The effect size was calculated by converting partial eta square to Cohen’s d (d) quantify meaningful differences in the data with demarcations of trivial (< 0.2), small (0.2–0.59), medium (0.60–1.19), large (1.2–1.99), and very large (≥ 2.0) being used in all statistical tests, the significance level is *p* < 0.05 [33].

To assess test–retest reliability thresholds for the intraclass correlation coefficient (Cronbach’s ICCs) were computed using a two-way mixed model. Regarding injury data, we used Shapiro–Wilk tests which indicated the non-normality of the distributions of injuries between the STG and CG for type of injury (muscle strains/contractures, sprains/ligament injury, contusions/hematoma/tissue bruising, fracture/dislocation, laceration, central/peripheral nervous system, tendinosis joint injuries and other injuries), location of injury (upper body, lower body), and injury burden (minimal, mild, moderate, severe). Consequently, a non-parametric alternative, the Mann–Whitney U test, was used to investigate differences between both groups.

## Results

### Training

All participants were administered their assigned training schedules, and there were no reports of injuries related to the training or assessments. Three athletes in the CG were excluded from the analysis (i.e., pre-protocol analysis) due to injuries unrelated to testing, hence the groups ultimately included 13 players in both STG and the CG.

### Anthropometric and Body Composition Characteristics

The baseline anthropometric measures were not significantly different between STG and CG *(*Table 6*)*. A significant main effect of time occurred during training for body mass (*p* < 0.001; d = 2.50), BMI (*p* < 0.001; d = 2.50), % body fat (*p* = 0.011; d = 1.13), lean mass (*p* < 0.001; d = 2.36), lower limb length (*p* = 0.001; d = 1.58). There were group-by-time interactions for body mass (*p* = 0.009; d = 1.17), BMI (*p* = 0.013; d = 1.09), and lean muscle mass (*p* = 0.008; d = 1.18). However, there were no group-by-time interactions for the other anthropometric parameters such as height, percentage of body fat, sitting height and lower limb length (0.078 < *p* < 0.265; 0.43 < d < 0.75). Post hoc tests revealed pre-to-post test increases in anthropometric measurements for lean muscle mass (*p* = 0.008; d = 1.18), body mass (*p* = 0.009; d = 1. 17), and BMI (p = 0.013; d = 1.09) in the STG.

**Table 6:** Effects of 12 weeks of strength training on anthropometrics and body composition of female soccer players (means ± SDs)

Variables	Group	Pre	Post	% Change	ANOVA *p*-value (Cohen’s d)		
					Main effect	Interaction	
					Time	Group	Group × Time
Body mass (kg)	CG	57.82 ± 7.71	58.75 ± 7.41	1.62	< 0.001 *(2.50)	0.590(0.22)	0.009*(1.17)
	STG	55.25 ± 8.60	57.82 ± 8.99	4.66			
Height (cm)	CG	163.92 ± 6.64	164.00 ± 6.58	0.05	0.112(0.67)	0.939(0.00)	0.265(0.43)
	STG	163.96 ± 7.48	164.38 ± 7.39	0.26			
BMI (kg.m^−2^)	CG	21.50 ± 2.37	21.89 ± 2.38	1.83	< 0.001*(2.50)	0.389(0.35)	0.013*(1.09)
	STG	20.31 ± 2.95	21.31 ± 2.63	4.92			
%Body fat	CG	26.94 ± 2.62	26.59 ± 1.99	− 1.30	0.011*(1.13)	0.082(0.74)	0.141(0.62)
	STG	25.45 ± 3.37	24.25 ± 2.93	− 4.72			
Lean muscle mass (kg)	CG	43.79 ± 4.53	44.08 ± 4.361	0.66	< 0.001*(2.36)	0.857(0.063)	0.008*(1.18)
	STG	43.16 ± 5.00	44.02 ± 5.21	2.01			
Sitting height (cm)	CG	82.73 ± 3.03	83.50 ± 3.05	0.93	0.188(0.552)	0.780(0.10)	0.079(0.74)
	STG	83.50 ± 3.15	83.50 ± 3.15	− 0.14			
Lower limb length (cm)	CG	86.15 ± 4.06	86.54 ± 4.16	0.45	0.001*(1.58)	0.578(0.22)	0.078(0.75)
	STG	84.81 ± 5.07	85.89 ± 4.83	1.27			

*CG* control group, *STG* strength-training group, *BMI* body mass index

*: Significantly different, *p* < 0.05

### Physical Fitness Tests

Baseline measures of physical fitness were similar in STG and CG (Tables 7, 8). There were main time effects for sprint (5-m,10-m), SJ, CMJ, 5JT, YYIRTL1, T-test (with and without ball), RSSA (mean, best, total and decrement), absolute and relative 1-RM (lat pull-down, bench press and leg press; *p* < 0.05; 0.58 < d < 2.55). Moreover, there were also main group effects for sprint (5 m, 10 m), and 30 m, YYIRTL1, T-test with and without ball, RSSA (mean, total, and decrement), relative and absolute 1-RM (lat pull-down, bench press, leg press; *p* < 0.05; 0.676 < d < 2.79).

**Table 7:** Effects of 12 weeks of strength training on physical fitness (means ± SDs)

Variables	Group	Pre	Post	% Change	ANOVA p-value (Cohen’s d)		
					Main effect		Interaction
					Time	Group	Group × Time
Sprint 5-m (s)	CG	1.12 ± 0.09	1.19 ± 0.08	6.25	0.005*(1.27)	0.034*(0.92)	0.085(0.73)
	STG	1.09 ± 0.05	1.11 ± 0.04	1.83			
Sprint 10-m (s)	CG	1.99 ± 0.12	2.06 ± 0.11	3.52	0.002*(1.40)	0.008*(1.18)	0.287(0.44)
	STG	1.91 ± 0.07	1.94 ± 0.05	1.57			
Sprint 30-m (s)	CG	5.02 ± 0.28	5.14 ± 0.28	2.39	0.123(0.65)	0.003*(1.38)	0.113(0.67)
	STG	4.80 ± 0.19	4.79 ± 0.15	− 0.21			
Squat jump (cm)	CG	23.52 ± 3.32	24.08 ± 3.45	2.38	0.015*(1.06)	0.767(0.13)	0.083(0.73)
	STG	22.61 ± 3.60	25.69 ± 3.29	13.62			
CMJ (cm)	CG	24.79 ± 3.16	25.28 ± 2.90	1.98	0.001*(1.63)	0.868(0.063)	0.005*(1.27)
	STG	22.85 ± 3.29	26.84 ± 3.60	17.46			
5JT (m)	CG	9.07 ± 0.62	9.54 ± 0.72	5.95	< 0.001*(1.86)	0.198(0.54)	0.616(0.21)
	STG	9.35 ± 0.83	9.95 ± 0.71	6.42			
YYIRTL1 (m)	CG	595.38 ± 203.33	740.00 ± 319.69	24.29	0.027*(0.99)	< 0.001*(1.31)	0.186 (0. 38)
	STG	890.77 ± 330.92	1283.08 ± 436.11	44.04			
T-test with ball (s)	CG	13.72 ± 0.99	12.89 ± 0.54	− 6.05	< 0.001*(2.34)	< 0.001*(2.01)	0.166(0.58)
	STG	12.29 ± 1.40	10.91 ± 0.92	− 11.23			
T-test without ball (s)	CG	12.63 ± 0.50	12.08 ± 0.44	− 4.35	< 0.001*(2.55)	< 0.001*(2.79)	0.246(0.49)
	STG	11.59 ± 0.62	10.78 ± 0.49	− 6.99			
RSSA _Best_ (s)	CG	3.50 ± 0.21	3.52 ± 0.26	0.57	0.052(0.57)	0.183(0.39)	0.103(0.48)
	STG	3.34 ± 0.16	3.54 ± 0.13	5.99			
RSSA _Mean_ (s)	CG	3.78 ± 0.21	3.82 ± 0.21	1.06	0.050*(0.58)	< 0.001*(1.84)	0.007*(0.81)
	STG	3.60 ± 0.14	3.37 ± 0.13	− 6.39			
RSSA _Total_ (s)	CG	22.68 ± 1.27	22.90 ± 1.23	0.97	0.104 (0.68)	0.002*(1.381)	< 0.001*(1.902)
	STG	21.65 ± 0.87	21.21 ± 0.77	− 2.03			
RSSA _Decrement_ (%)	CG	8.48 ± 2.50	8.19 ± 2.41	− 3.42	0.036*(0.95)	0.042*(0.92)	0.082(0.78)
	STG	7.73 ± 3.82	4.92 ± 2.19	− 36.35			

*CG* control group, *STG* Strength-training group, *SJ* squat jump, *CMJ* countermovement jump, *5JT* five-jump test, *YYIRTL1* yoyo intermittent recovery test level1, *RSSA* repeated-shuttle-sprint ability

*p ≤ 0.05

**Table 8:** Effects of 12 weeks of strength training on markers of muscle strength (means ± SDs)

Variables		Group	Pre	Post	% Change	ANOVA *p*-value (Cohen’s d)		
						Main effect		Interaction
						Time	Group	Group × time
Lower limbs	1-RM bench press (kg)	CG	19.18 ± 3.74	21.98 ± 3.56	14.62	< 0.001*(5.11)	0.002*(1.43)	< 0.001*(2.59)
		STG	21.99 ± 3.80	30.54 ± 5.69	38.85			
	1-RM bench-press relative	CG	0.34 ± 0.09	0.38 ± 0.08	11.37	< 0.001*(4.55)	0.002*(1.42)	< 0.001*(2.39)
		STG	0.40 ± 0.05	0.53 ± 0.08	32.34			
Trunk	1-RM lat-pull-down (kg)	CG	35.59 ± 4.58	37.73 ± 4.53	6.02	< 0.001*(2.72)	0.052(0.83)	< 0.001*(1.68)
		STG	35.62 ± 4.20	44.72 ± 5.99	25.55			
	1-RM lat-pull-down relative	CG	0.63 ± 0.14	0.66 ± 0.13	3.95	< 0.001*(2.08)	0.023* (0.676)	0.091(0.498)
		STG	0.65 ± 0.06	0.78 ± 0.08	19.48			
Upper limbs	1-RM leg-press (kg)	CG	51.78 ± 11.12	66.96 ± 10.70	29.33	< 0.001*(4.53)	< 0.001* (1.130)	0.068(0.538)
		STG	62.39 ± 20.20	96.89 ± 27.33	55.29			
	1-RM leg-press relative	CG	0.92 ± 0.28	1.16 ± 0.24	25.79	< 0.001*(4.39)	0.002*(1.41)	< 0.001*(1.72)
		STG	1.11 ± 0.23	1.66 ± 0.33	49.42			

*CG* control group, *STG* Strength-training group, *1-RM* one repetition maximum

*p < 0.05

There was a significant group-by-time interaction for CMJ, RSSA (mean, total) performances, absolute (bench press, lat pull-down), and relative 1-RM (bench press, leg press; 0.001 < *p* < 0.007; 0.81d < 2.59).

Post hoc tests indicated pre-to-post improvements with advantages for the STG related to CMJ (p = 0.015, d = 1.22), RSSA means (*p* = 0.007, d = 1.33), absolute 1-RM bench press (*p* < 0.001, d = 1.994), relative 1-RM bench press (*p* < 0.001, d = 1.662), absolute 1-RM lat pull-down (*p* < 0.001, d = 1.876), relative 1-RM leg press (*p* < 0.001, d = 2.022).

### Training Load

The internal load (RPE) was found to be similar to STG and CG. The STG had a load of 1158.4 ± 67.7AU, while the CG had a load of 1188.82 ± 44.1 AU, with no differences between the two groups for total training volume over the 12-week ST period (*p* = 0.205, d = 0.25).

### Injury Occurrence

Only non-contact injuries were taken into account in our analysis. A total of 13 non-contact injuries were recorded over the soccer season (from September to May) in the involved experimental groups (Table 9). There were significantly more non-contact injuries in the CG (11 injuries) than the STG (2 injuries) (*p* = 0.003; d = 1.31), with the majority of injuries occurring during the follicular phase of the players' menstrual cycles in both groups. More specifically, there were 11 injuries in the follicular phase and 2 in the luteal phase (*p* = 0.013; d = 1.05). Furthermore, there were significantly more injuries reported in the late follicular phase (*p* = 0.026; d = 0.91) in the CG (63.6%) compared to the STG (50%). The majority of injuries in both groups were located in the lower body (91%), with some (9%) in the upper body. Twelve lower limbs injuries and one low back injury were noted *(*Tables 9, 10).

**Table 9:** Types of non-contact injuries

Injury type	Group	Non-contact injuriesduringmenstrualcycle				
		Injuries recorded	Early follicular phase	Late follicular phase	Mid-luteal phase	Other phases
Muscle strains/contractures	CG	2 (18.2%)	0 (0%)	2 (18.2%)	0 (0%)	0 (0%)
	STG	0 (0%)	0 (0%)	0 (0%)	0 (0%)	0 (0%)
Sprains/ligament ruptures	CG	8 (72.7%)	2 (18.2%)	4 (36.4%)	1 (9.1%)	1 (9.1%)
	STG	2 (100%)	1 (50%)	1 (50%)	0 (0%)	0 (0%)
Fracture/dislocation	CG	0 (0%)	0 (0%)	0 (0%)	0 (0%)	0 (0%)
	STG	0 (0%)	0 (0%)	0 (0%)	0 (0%)	0 (0%)
Low back pain	CG	1 (9.1%)	0 (0%)	1 (9.1%)	0(0%)	0 (0%)
	STG	0 (0%)	0 (0%)	0 (0%)	0 (0%)	0 (0%)
Total injuries	CG	11 (84.6%)	2 (18.2%)	7 (63.6%)	1 (9.1%)	1 (9.1%)
Total injuries	STG	2 (15.4%)	1 (50%)	1 (50%)	0 (0%)	0 (0%)
Total injuries		13 (100%)	3 (23.1%)	8 (61.5%)	1 (7.7%)	1 (7.7%)

*CG* control group, *STG* strength training group

**Table 10:** Non-contact injury data recorded during the different periods of the season

	Preseason period				Competitive period				Entire season			
	CG		STG		CG		STG		CG		STG	
*Injuries location*												
Upper body	0 (0%)		0 (0%)		1 (9%)		0 (0%)		1 (9%)		0 (0%)	
Lower body	0 (0%)		0 (0%)		10 (91%)		2 (100%)		10 (91%)		2 (100%)	
Total injuries	0 (0%)		0 (0%)		11 (100%)		2 (100%)		11 (100%)		2 (100%)	
*Injuries severity and number of absence days*												
*Minimal (1–3 days)*												
Number of injuries									7 (64%)		0 (0%)	
Days lost									17		0	
*Mild (4-7 days)*												
Number of injuries									2 (18%)		2 (100%)	
Days lost									11		14	
*Moderate (8–28 days)*												
Number of injuries									2 (18%)		0 (0%)	
Days lost									42		0	
*Severe (> 28 days)*												
Number of injuries									0 (0%)		0 (0%)	
Days lost									0		0	
*Total days lost*												
									70		14	
*Exposure time(a)*												
									4191		4191	
Burden (b)									16.70		3.34	
*Incidence injury rate*												
Number of injuries (c)	0		0		11		2		11		2	
Exposure time training	630		630		3150		3150		3780		3780	
Exposure time match	59		59		352		352		411		411	
Exposure time total	689		689		3502		3502		4191		4191	
IIR (d) Injury Rate	0.00		0.00		3.14		0.57		2.62		0.48	
IRR (e)	0				0.18				0.18			

STG, strength training group; CG, control group; (a) Exposure time calculated using the number of female players per training session (around 14). Number of training sessions (around 180 for the examined period. including 30 training sessions during preseason period) and number of played matches (28 matches for the studied period. including 4 matches during preseason period); (b) burden = 1000 × (∑ days absent/∑ exposure hours) [32] (c) Number of injuries was recorded during training and matches. (d)IIR (Incidence injury rate) was calculated. IIR = 1000 × (∑ injuries/∑exposure hours) [32]. Season IIR (Incidence injury rate) including preseason and competition periods. (e) IRR, Injury rate ratio = injury incidence of STG divided by the injury incidence of CG

Regarding injury types, ligament injuries were the most common in both groups, with eight injuries (72.7%) in the CG and two injuries (100%) in the STG (*p* = 0.084; d = 0.68). In addition, two muscle injuries were recorded for the CG (18.2%) while the STG experienced no muscular injuries, (*p* = 0.093; d = 0.66). The one case of low back pain was observed in CG.

In terms of athlete availability and injury severity, 11 injuries were recorded for the CG during the season, which accounted for a total loss of 70 days, resulting in absences from both, training and the regular competitive season. Within the 11 injuries, seven were classified as minimal injuries (1–3 days loss), two were mild injuries (4–7 days loss), two were moderate injuries (8–28 days loss). No severe injuries were reported (+ 29 day) [18]. In comparison, the two injuries in the STG accounted for a total loss of 14 days, with both of these cases classified as mild injuries (4–7 days loss). The injury burden was significantly greater in the CG (burden = 16.7 days absent per 1000 h) compared to the STG (burden = 3.3 days absent per 1000 h) (*p* = 0.011; d = 1.07) *(*Table 10*).*

The IIR during the season was 2.62 per 1000 h of exposure in the CG and 0.48 per 1000 h of exposure in the STG, resulting in an injury rate ratio (IRR) of 0.18. However, during the competitive period, the IIR was 3.14 in the CG and 0.57 in the STG, with an IRR of 0.18.

## Discussion

This study investigated the effects of 12 weeks of ST on anthropometric and body composition characteristics, physical fitness, and strength performance indicators in North African elite female adolescent soccer players and the occurrence of non-contact injuries.

### Anthropometric and Body Composition Characteristics

The ST protocol in our study increased significantly overall body mass as well as lean muscle mass of elite female soccer players. Several studies reported that ST improves body composition in female athletes by increasing lean muscle mass and decreasing body fat percentage. For instance, a recent systematic review of 33 studies indicated that resistance training (RT), or combinations of RT with other strength-dominated exercise types, improved body composition and muscle morphology in elite female athletes [11].

In support of this, Roso-Moliner et al. [34] reported that a 10-week neuromuscular training program with three weekly sessions reduced fat mass and increased muscle mass in highly trained female soccer players, suggesting that neuromuscular training promotes muscle hypertrophy and improves body composition [35]. Additionally, studies by Lesinski et al. [23] on the effects of long-term ST on body composition in U17 female soccer players demonstrated increased total and segmental lean body mass. In another study, the researchers could not detect any significant differences in the body composition of female soccer players with a mean age of 22.1 ± 1.1 years following 8 weeks of ST [4]. This discrepancy in findings can most likely be explained by the difference in the duration of the exercise regimes and the lower body fat percentage of the elite female soccer players in our study.

### Physical Fitness Tests

#### Maximal Dynamic Strength

We found a significant group-by-time interaction in maximal strength (i.e.; 1-RM) in the lower and upper limbs and the trunk in the STG, with no increases observed in the CG. Our results are supported by recent studies in young female soccer players confirming that sports requiring muscle power for optimal performance rely on ST, and that ST may be an effective method for improving both, maximal strength and jump performance [7, 35].

ST increases muscle strength in young females, and ST programs lasting at least 8 weeks, with two 40 min-sessions per week, may be the most effective approach [36]. Incorporating ST into regular soccer training improved maximal strength in both the upper and lower body of 15-year-old soccer players [7]. Implementing a ST program, leads to improvement in 1-RM of squat exercise ranging from 11 to 52% [37]. Recent studies confirmed the efficacy of ST in improving maximal strength in young elite female soccer players [17, 23, 38]. ST over one season improved muscle power and 1-RM leg extensor strength in young elite female soccer players [23]. Similarly, the importance of an in-season ST program for developing strength was confirmed in adolescent female soccer players, with increases of 24% and 34% in 3-RM hip thrust and squat respectively [38]. Strength increases were influenced by the athletes’ lack of ST experience [38]. Increases in maximal squat strength (1-RM) occurred after 12 weeks of ST (3 sessions per week) in young female soccer players [17]. In our study, enhanced maximal strength (i.e.,1-RM) could be due to improvements in motor unit recruitment and firing frequency. Indeed, the neural adaptations in female athletes, particularly in upper-body or lower-body muscles, increase during the initial weeks of ST, leading to higher relative strength gains. Females can experience improved strength and muscle mass during progressive ST [11].

#### Muscle Power

##### Squat Jump, Countermovement Jump, and Five Jump Tests

The CMJ test is a standard measure of lower body power [7]. We recorded a significant group-by-time interaction in CMJ in favor of the STG, findings which agree with recent reports confirming that ST may be the most efficient method for improving both maximal strength and jump performance [7, 39]. Moreover, ST either with or without external loading, improves physical fitness and body composition in young soccer players. A 15-week ST program increases the jumping ability of young male soccer players [40]. Likewise, Lesinski et al. [23] reported that power training performed throughout the season (at 50–95% of the 1-RM) enhanced CMJ performance in elite young female soccer players, while a study by Fernandez Ortega et al. [17] added that 12 weeks (3 sessions/week) of strength and power training yield enhancement in CMJ and SJ. Additionally, Fischerova et al. [41] reported that six weeks of ST (two sessions per week, at 50–85% of 1-RM) led to an 11% increase in CMJ in highly trained female soccer players.

According to McKinlay et al. [39], ST may be the most successful method for raising maximum strength and jump performance. A recent study indicates that compound multi-joint ST, as used in our study, improves jumping ability and explosive power [7]. In comparison to other training methods, our study and those of others [42], indicate that squats (or half-squat exercises) may be especially useful for improving soccer players jumping abilities [7]. A systematic review and meta-analysis supported the view that ST improves physical fitness tests and sport-specific performance in young athletes (6–18 years old) [23]. Other studies of young female soccer players indicated that both back squats and hip thrusts probably improved vertical jump height (5–10%) with no changes in horizontal jump [38], and aligns with our findings that the 5JT caused no improvements after 12 weeks of ST for female soccer players. Similarly, Contreras et al. [43] reported that front squat training was marginally more effective than hip thrusts in improving vertical jumping ability, but that neither exercise affected horizontal jumping. Greater improvements in CMJ with weightlifting were caused by increased leg stiffness, decreased co-activation of the antagonist muscle (rectus femoris), and higher activation of the agonist muscle [11].

#### Linear Sprint and Change-of-Direction Speed

Participants exposed to the ST program experienced no improvements in the assessments of their linear speed (sprint 5-m, 10-m, 30-m) or change-of-direction (CoD). Our findings are in agreement with recent studies reporting no improvements in linear sprint or CoD after an in-season ST program for young female soccer players [38]. A recent study reported that 6 weeks of in-season hip thrust vs. back squat training in female adolescent high school players did not improve their linear sprint performance [38]. High-load repetitions (70–95% 1-RM) are frequently utilized in studies with young soccer players to increase strength and power [17]. However, high-load ST can cause extreme exhaustion, making it challenging for athletes to engage in productive ball practice right immediately after this kind of training. Furthermore, because the neural system is unable to regulate the levels of strength or muscle mass gained during extremely quick movements, training with heavy loads is not transferable to sprint velocity performance [17].

Performances in sprinting (5 and 20-m) and CoD decrease during both mid-season and post-season compared to the pre-season [2]. In contrast, an in-season 8-week combined strength and power training program improved sprint and CoD performance in adolescent female soccer players [12]. Previous studies demonstrated improved speed performance over the entire season after ST in elite young female soccer players [23], while weekly strength and conditioning sessions improved sprint-related performance in young female soccer players over 3 years [44]. Moreover, reactive strength increases the speed and ability to change directions in female soccer players [2]. It is important to note, these studies used ST for longer periods than in our study.

The lack of significant group-by-time interactions in linear speed and CoD tests is contrary to our hypothesis and could be attributed to several factors, including the duration and intensity of the ST program, the initial level of physical fitness among the athletes, and the specific exercises incorporated into the program [34]. Our results are in agreement with the meta-analysis of Nuñez et al. [45] indicating that ST applied during the in-season period of soccer players may be less effective than during the pre-season. Systematic reviews did not identify one strength or power measure that was consistently associated with COD performance in all included trials, possibly because strength and power exercises are often performed bilaterally in a vertical plane [23]. In contrast, COD tasks mainly involve horizontal movements and mostly unilateral limb accelerations [23]. Athletes require more than ST alone to maximize the development of linear or change-of-direction speed [38]. Our findings could be explained by findings that high-velocity, low-load ST (30–60% 1-RM) produces better results in sprint ability and maximal aerobic speed compared to low-velocity, high-load training (70–95% 1-RM) [17].

#### Soccer-Specific Performance

##### RSSA and YYIRTL1

There were significant main effects of time (d = 0.99) and group (d = 1.31) for YYIRTL1 performance, where pre-post test performances were improved in CG (+ 24%) and STG (+ 44%). However, there were no significant group-by-time interaction after the 12 weeks ST. Our results are in line with other studies indicating that ST with and without load has no effect on aerobic capacity [40]. Additionally, significant main effects of time were found for RSSA_Mean_ (d = 0.58), RSSA_Best_ (d = 0.57), RSSA_Total_ (d = 0.68), and RSSA_Decrement_ (d = 0.95). Interestingly our results indicated significant group-by-time interactions for RSSA _Mean_ and RSSA_Total_, (-6.39% and -2.03%) in favor of the STG, and are supported by other studies confirming that ST improved RSSA_Best_ (− 2.90 ± 2.1) and RSSA_Mean_ (− 2.61 ± 2.8) in young elite soccer players [46]. Soccer performance is improved by ST [47], and there is a positive correlation between maximal squat strength, sprinting, and jumping in elite soccer players suggesting that strengthening quadriceps muscles improves soccer performance [47]. Enhancing an athlete's strength can improve both initial acceleration and maximal sprint speed; the improvement in initial acceleration can be particularly advantageous in soccer, where there are approximately 60 accelerations per match [48]. Consequently, increased muscle strength can enhance acceleration speed, and impulsivity [49].

Our study demonstrates improvements in maximum strength variables measured on the leg press device, indicating that the ST component of the program had a significant impact on lower limb power, which is crucial for soccer players in activities such as sprinting [7]. Impulse, which is the product of force by time, has an important role in acceleration. However, since time is limited during the ground contact phase, it is essential to maximize force production within this time frame [48]. Maximizing initial acceleration requires maximal strength. Ground contact times greater than 200 ms allow for greater force transfer [49].

### Injury Occurrence

Soccer is a high intensity, contact sport that carries a significant risk of injury [1]. Exposing soccer players to sudden and significant changes in athletic load increases the risk of injury in elite players of both sexes [13]. Men's professional soccer players experience 10–35 injuries per 1000 h, compared to 9.1–24 injuries per 1000 h for professional female soccer players [13]. While the overall injury incidence is higher in elite male soccer players than in their female counterparts, women appear to have a significantly higher proportion of severe injuries (absence of > 28 days) [13]. Injury risk factors in elite female soccer players are multifactorial and complex, with various intrinsic factors such as hormonal fluctuations during the menstrual cycle potentially influencing the risk of injury. Indeed, injuries are a frequent occurrence in adolescent soccer players, especially throughout periods of rapid growth and maturation [50], whereas they have scarcely been investigated in elite young female soccer players [51].

#### Location of Injuries

In our study, non-contact injuries in the lower body accounted for 76.9% in CG and 15.4% in STG of all injuries. A recent systematic review and meta-analyses reported that the highest overall 'time loss' IIRs in female soccer players was due to injuries in lower limbs (4.54/1000 h; 95% CI 3.97–5.19) [52]. Other studies indicated that 85% of the injuries were to the lower extremities (most commonly to the thigh, knee, and ankle) [10], with similar findings in young) [51] and adults [53] elite female soccer players.

#### Type of Injuries

Non-contact injuries in the CG were higher in ankle; there were 8 (61.5%) ankle sprains, whereas ankle sprains in the STG accounted for 15.4% of the total injuries during the season. Non-contact muscle strains accounted for 15.4% of injuries in CG and included 1 calf contracture and 1 hamstring-elongation. Even though hamstring injuries are the most common muscle injuries in young soccer players [14], there were no muscle injuries in the STG.

Our study findings are in accordance with research results from previous studies conducted with elite female soccer players. A recent systematic review and meta-analysis in adult elite female soccer showed that the most common injury was blunt soft tissue damage (contusions and hematomas; 44%, 165/378), followed by ligament sprains (25%, 96/378) and muscle strains (10%, 38/378) [53].

Likewise, our findings also agree with the higher proportion of injuries mainly in knees and ankles (30.4% and 17.9 respectively) reported in elite female Spanish soccer players; moreover, Spanish female soccer players commonly suffer from ligament injuries and contusions, which account for 65.8% of all injuries, with ligament injuries being the most prevalent in this category (38.5 of total injuries) [54].

A recent systematic review indicates that injuries to the lower limbs of adult female soccer players were highest (4.8 injuries/1000 h exposure), with the most common injury types being muscle/tendon (1.8 injuries/1000 h exposure) and joint (non-bone) and ligament (1.5 injuries/1000 h exposure), which were often associated with traumatic events [55]. Additionally, another contemporary systematic review of injuries in young male and female soccer players revealed that minor injuries were the most common in both sexes with the lower limb having the highest incidence rate. In Females, joint/ligament injuries were the most common [50].

#### Mechanisms of Injuries

There were more non-contact injuries in the CG than STG in our study, which may be related to the risk factors for injuries in soccer caused by inadequate strength levels of the athletes [14]. In this regard, ST programs reduce muscle imbalances, which are often associated with an increased risk of injury and can contribute to injury prevention by stabilizing lower limb muscles and increasing the force of a muscle during an eccentric action [14]. The mechanisms of injuries in female soccer players include biomechanical, neuromuscular, and environmental factors. Hormonal (such as progesterone and estrogen) changes during the menstrual cycles have been associated with ligamentous laxity, impaired neuromuscular function. These hormonal changes could affect a female athlete's ability to maintain both passive and active knee stability [54].

Intrinsic risk factors for soccer players include reduced knee alignment, such as increased dynamic valgus and high abduction loads, and decreased knee and hip flexion angles during landing furthermore, decreased lower body strength, a lower hamstring/quadriceps (H/Q) ratio during the concentric action generalized joint hypermobility (laxity), and particularly knee hyperextension elevates the risk of lower limb injury [4]. Strengthening core muscles with ST helps to maintain correct body tension when jumping for a heading duel and reduces injury risks [13]. The use of injury prevention programs to enhance movement proficiency and physical fitness in young soccer has decreased the incidence of moderate and severe non-contact injuries in children and teenagers [50].

#### Severity of Injuries

Injury severity was classified according to the number of days missed from training or matches as described by Fuller et al. [32]: minimal (≤ 3 days), mild (4–7 days), moderate (8–28 days), and severe (> 28 days). The CG in our study experienced more lost days (16.7 days absent/1000 h) than STG (3.34 days absent/1000 h) and most injuries appeared to be minimal (1–3 days) with 64% in CG with no minimal injuries in the STG. These findings complement earlier studies indicating that minimal injuries were the most common in females followed by moderate [50]. Our data agree with several studies on the importance of ST in reducing the overall rate of injuries [18] and the days’ absence of elite young soccer players [14] An earlier study reported that half the injuries in female soccer players were categorized as severe [54].

#### Incidence Injury Rates

The IIR during the season (including both match and training exposure) was 2.62 per 1000 h of exposure in the CG and 0.48 per 1000 h of exposure in the STG. The injury rates in the competitive period, were 3.14 injuries per 1000 h and 0.57 injuries per 1000 h in CG and STG, respectively. Moreover, our findings indicate IIR ratios of 0.18 for the entire season and 0.18 during the competitive season. These results highlighted the importance of ST in reducing injuries in young female soccer players regardless of the time (pre-season vs. competitive games). Our findings agree with previous studies reporting that ST prevents and reduces injuries in both male and female soccer players [14]. Likewise, the high IIRs and probability scores found in soccer players in the meta-analysis of Robles-Palazón et al. [50] reinforce the need for implementing injury prevention programs in young soccer players to provide the robustness and readiness needed for competitive play.

#### Menstrual Cycle and Injuries

Menstrual cycles can potentially alter injury risk in female athletes, as changes in reproductive hormones could impact musculoskeletal tissues such as muscles, tendons, and ligaments [56]. We recorded 13 non-contact injuries in our study, with 84.6% of all injuries occurring in the follicular phase (23.1% in the early follicular phase; 61.5% in the late follicular phase) of the menstrual cycle of female players, including 8 ligament injuries (6 in the CG and 2 in the STG), and only in the CG, we recorded 2 injuries of muscular origin and 1 low back injury.

A recent study reported that the menstrual cycle phases do not affect strength and power performance in elite team athletes [57]. Furthermore, previous studies confirmed that muscle and tendon injuries are approximately twice as common in the late follicular phase, when estrogen concentrations are highest, compared to the follicular and luteal phases [57]. This occurs despite some findings that estrogen is negatively associated with tendon stiffness and that tendon stiffness is lowest in the late follicular phase [56]. Another recent study reported that the risk of injuries during menstrual cycles is increased by hormonal fluctuations, especially during ovulation [58].

## Practical Applications

Current approaches in ST support the use of ST machines as being safe for elite young female soccer players with no previous experience in weightlifting. Since research on ST programs and evidence is constantly evolving, new ideas and recommendations are important guiding principles for future studies. To that end, our findings suggest the application of a familiarization/technique phase before gradually increasing volume and intensity in subsequent ST phases. We emphasize the importance of qualified guidance, effective supervision, and gradual progression of volume and intensity, as in adult training principles. Moreover, it is important to provide adequate supervision, short-term planning, long-term variations, individualized loads based on %RM, and adequate rest periods. This approach will prevent acute and overuse injuries for the ST intervention and the soccer-specific training.

## Study Limitations

There are methodological limitations related to the current study that must be acknowledged. First, the females used here in were eumenorrheic and ovulatory prior to the study beginning (i.e., normally menstruating). Second, a lack of control on side factors (“events of daily living”) that each subject experienced outside training/study participation. Each of these factors could serve as confounding elements within our study. However, we feel it is important to denote that our study has several strengths too; such as, (1) there was a careful control and monitoring of training sessions, (2) extensive evaluative assessments of performance, and (3) a thorough and detailed appraisal of injuries occurrences in the subjects.

## Conclusions

This study investigated the effects of in-season ST on physical fitness and injury occurrence in young North African female soccer players. Our results indicate that soccer training combined with 12 weeks of ST and 2 weekly sessions, even when implemented during the season, improves anthropometric and body composition characteristics as well as measures of physical fitness. In addition, the applied ST regime reduced the incidence of non-contact injuries in young female soccer players.

## Data Availability

All data supporting the findings of this study are available upon a reasonable request to the corresponding authors.

## Abbreviations

1-RM: One-repetition-maximum
5JT: Five jump test
AU: Arbitrary units
ACL: Anterior cruciate ligament
ANOVA: Analysis of variance
BW: Body weight
CG: Control group
CMJ: Countermovement jump
CoD: Change-of-direction
d: Cohen’s d
DM: Duration of the match in minutes
E2: 17-estradiol: Estrogen
FIFA: Fédération Internationale de Football Association
ICC: Intra-class correlation coefficients
IIR: Incidence injury rate
IRR: Injury rate ratio
Nb: Number
NM: Number of team matches played
P4: Progesterone
PHV: Peak height velocity
PM: Number of players in the team
RPE: Ratings of perceived exertion
RSSA: Repeated shuttle sprint ability
SD: Standard deviations
SJ: Squat jump
sRPE: Session-rating of perceived exertion
ST: Strength training
STG: Strength training group
YYIRTL1: Yo-Yo intermittent level1
